# Characterization of a thermophilic cytochrome P450 of the CYP203A subfamily from Binh Chau hot spring in Vietnam

**DOI:** 10.1002/2211-5463.13033

**Published:** 2020-11-30

**Authors:** Kim‐Thoa Nguyen, Ngoc‐Lan Nguyen, Mohammed Milhim, Van‐Tung Nguyen, Thi‐Hong‐Nhung Lai, Huy‐Hoang Nguyen, Thi‐Thanh‐Xuan Le, Thi‐Tuyet‐Minh Phan, Rita Bernhardt

**Affiliations:** ^1^ Institute of Biotechnology Vietnam Academy of Science and Technology Hanoi Vietnam; ^2^ Graduate University of Science and Technology Vietnam Academy of Science and Technology Hanoi Vietnam; ^3^ Institute of Genome Research Vietnam Academy of Science and Technology Hanoi Vietnam; ^4^ Department of Biochemistry Saarland University Saarbrucken Germany

**Keywords:** Binh Chau hot spring, CYP203A subfamily, cytochrome, DNA metagenome, P450‐T2, thermophilic P450

## Abstract

Cytochromes P450 (CYPs or P450s) comprise a superfamily of heme‐containing monooxygenases that are involved in a variety of biological processes. CYPs have broad utilities in industry, but most exhibit low thermostability, limiting their use on an industrial scale. Highly thermostable enzymes can be obtained from thermophiles in geothermal areas, including hot springs, offshore oil‐producing wells and volcanoes. Here, we report the identification of a gene encoding for a thermophilic CYP from the Binh Chau hot spring metagenomic database, which was designated as P450‐T2. The deduced amino acid sequence showed the highest identity of 73.15% with CYP203A1 of *Rhodopseudomonas palustris*, supporting that P450‐T2 is a member of the CYP203A subfamily. Recombinant protein expression yielded 541 nm. The optimal temperature and pH of P450‐T2 were 50 °C and 8.0, respectively. The half‐life of P450‐T2 was 50.2 min at 50 °C, and its melting temperature was 56.80 ± 0.08 °C. It was found to accept electrons from all tested redox partners systems, with BmCPR‐Fdx2 being the most effective partner. Screening for putative substrates revealed binding of phenolic compounds, such as l‐mimosine and emodin, suggesting a potential application of this new thermophilic P450 in the production of the corresponding hydroxylated products.

## Graphical abstract

A predicted alkali‐thermophilic cytochrome P450‐T2 from the DNA database of Binh Chau hot spring was synthesized, expressed and purified. The properties of the enzyme, including the optimum pH and temperature, melting temperature and half‐life at 50 °C were characterized. P450‐T2 accepts electrons from the BmCPR‐Fdx2 system. l‐Mimosine and emodin may be its putative substrates.

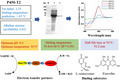

**Abbreviations**
Adx: adrenodoxin
CYP: cytochrome

Cytochromes P450 (CYPs or P450s) comprise a superfamily of heme‐containing monooxygenases that are involved in a variety of biological processes, such as carbon assimilation, biosynthesis of endogenous compounds, as well as terpenoids, biodegradation, xenobiotic detoxification and drug metabolism [1]. CYPs are ubiquitous in nature; however, most of them are less thermostable enzymes, preventing their practical applications on a larger scale [2]. Enzymes with unique characteristics, such as alkalinity, acidity, thermostability and cold activity, are found in extreme ecosystems. In particular, thermostable enzymes are obtained from thermophiles in geothermal areas, including hot springs, offshore oil‐producing wells and volcanoes [3, 4]. Thermostable P450s can serve as potential biocatalysts for the synthesis of valuable organic compounds. Research has been focused on identifying such thermostable P450s from nature, as well as using protein engineering to enhance their stability at high temperature [5, 6, 7]. To date, 11 thermostable CYPs have been derived from culturable species, namely, CYP119A1, CYP119A2, CYP174A1, CYP175A1, CYP231A2, CYP154H1, CYP116B29, CYP116B46, CYP116B63, CYP116B64 and CYP116B65 [8, 9]. Moreover, the bacterial CYP102A2 subfamily and the human CYP2B subfamily were rationally evolved for greater thermostability [8].

Although the demand for new enzymes is increasing, the biocatalytic capabilities of most microorganisms remain unexplored, because ~99% of them are unknown and cannot be cultured under laboratory conditions [10]. Metagenomic shotgun sequencing has emerged as a powerful tool to explore the composition and function of complex microbial populations residing in extreme environments. In a previous study, we analyzed the metagenomics of the Binh Chau hot spring in Vietnam and obtained 68 putative ORF encoding CYPs P450 belonging to 36 specific groups [11]. Based on the prediction of the melting temperature (*T*_m_) index, we aimed to identify a thermophilic CYP P450 enzyme with a novel sequence from the Binh Chau hot spring and were successfully able to identify and express a novel isoenzyme with activity toward testosterone [12]. In this study, we identified another sequence of a putative CYP P450, P450‐T2. It belongs to the CYP203A subfamily and was cloned and expressed in *Escherichia coli* strains. Purification and characterization of the P450‐T2 protein revealed that it is of moderate thermostability. Screening a limited library for potential substrates demonstrated type I binding of l‐mimosine and emodin. These results indicate the potential of thermophilic CYP P450 exploitation in geothermal areas via unculturable methods and provide new candidates for biotechnological application.

## Materials and methods

### Sequence analysis and gene synthesis

A 1188‐bp ORF, putatively encoding a CYP P450, namely, P450‐T2, of the Binh Chau metagenomic database, was selected for expression. *T*_m_ was predicted using a *T*_m_ predictor (http://tm.life.nthu.edu.tw/). The AcalPred tool [13] was used to predict the acidity and alkalinity of the enzyme. The nucleotide and amino acid sequences of P450‐T2 were deposited in GenBank with the accession number MT232929. The amino acid sequence identity between P450‐T2 and well‐annotated bacterial P450s in the CYP P450 database [14] was evaluated using BLAST + 2.9.0 [15]. The phylogenetic tree was constructed using the maximum‐likelihood method in mega x software (Institute of Molecular Evolutionary Genetics, University Park, PA , USA) [16]. Multiple sequences were aligned using clustal omega 1.2.4 (EMBL‐EBI, Cambridgeshire, UK) (https://www.ebi.ac.uk/Tools/msa/clustalo/). The nucleotide sequence of the P450‐T2 gene was synthesized and stored into pUC19 vector (PhuSa Biochem Ltd, Can Tho, Vietnam).

### Plasmid construction

Vector pET‐T2 was constructed by amplifying the P450‐T2 gene using the forward primer (5′‐GATCCATATGGGCCTTGGCAGCTTCCA‐3′) and the reverse primer (5′‐GATCAAGCTTA**GTGGTGATGGTGATGATG**CTGGGCCTTGAGCTGCAGCA‐3′) containing *Nde*I and *Hind*III sites (underlined), respectively. The bold letters in the reverse primer indicated the hexahistidine (His6)‐tag. The PCR product was digested subsequently with both corresponding enzymes and inserted into the opened *pET‐17b* vector (Novagen, Damstadt, Germany) by Fast link DNA ligation kit (Lucigen, USA). The clones were confirmed through PCR colony and sequenced subsequently by MWG Biotech (Ebersberg, Germany).

### Gene P450‐T2 expression

The plasmid pET‐T2 was transformed into *E. coli* JM109(DE3) (Promega Biosciences Inc. San Luis Obispo, CA, USA), *E* *. coli* BL21(DE3) and *E. coli* C43(DE3) (Novagen and inoculated in Luria Broth medium (BD, Sparks, MD, USA) containing ampicillin 100 µg·mL^−1^ overnight at 37 °C with shaking at 200 r.p.m. to obtain a preculture. A total of 2.5 mL of the preculture was transferred into a 2‐L baffled flask containing 250 mL Teffic Broth (TB) medium as a main culture. The main culture was inoculated at 37 °C with 150 r.p.m. shaking until the *A*_600_ value reached 0.8–1. At that point, the expression was induced by adding 1 mm IPTG and 0.5 mm δ‐aminolevulinic acid. The expression was carried out at 30 °C for 48 h with the same shaking speed.

### Protein purification

All the purification steps were carried out at 4 °C. The 1 L *E. coli* cell culture was centrifuged at 150 r.p.m. for 10 min. The pellets were disrupted by sonication in 20 mL of 50 mm Tris–Cl (pH 8.5), 1 mm EDTA, 100 mm NaCl, 0.1 mm dithioerythritol and 1 mm phenylmethanesulfonyl fluoride. The lysate was ultracentrifuged at     30 000 ***g*** for 30 min. The supernatant was loaded onto an affinity chromatography with an IMAC‐Ni^2+^ column (Bio‐Rad Laboratories GmbH, Feldkirchen, Germany). The column was treated with a washing buffer [50 mm potassium phosphate buffer (pH 7.2), 500 mm CH_3_COONa, 10% glycerol, 1.5% Tween 20, 0.1 mm phenylmethanesulfonyl fluoride, 0.1 mm dithioerythritol] supplemented with 50–200 mm imidazole. Elution procedure was carried out with the same buffer containing 400 mm imidazole at a rate of 1 mL·min^−1^. Fractions with *A*_417_:*A*_280 _> 1.6 were collected and dialyzed overnight to remove imidazole. Protein was concentrated by Centriprep (Merck Millipore, Darmstadt, Germany) with the pore size of 50 and 30 kDa down to 500 µL. The purified protein was stored at −80 °C. All samples were analyzed in 15% polyacrylamide gel SDS/PAGE [17].

### Spectrophotometric characterizations

UV‐visible spectra for the purified enzymes were recorded at room temperature on a double‐beam spectrophotometer (UV 2000PC; Shimadzu, Kyoto, Japan). The concentration of CYP was evaluated by CO‐difference spectrophotometer (Shimadzu) assuming Δε _(450–490)_ = 91 mm ^−1^ × cm^−1^ according to the method of Omura and Sato [18].

### CDs

The far‐UV spectra (195–260 nm) and the near‐UV spectra (300–450 nm) were scored with 4 and 20 µm P450‐T2, respectively, in 10 mm potassium phosphate buffer (pH 7.4) at 25 °C on a JASCO J715 spectropolarimeter (Jasco GmbH, Gross‐Umstadt, Germany).

### Optimal temperature and pH

The 5 µm purified enzyme was dissolved in 20 mm potassium phosphate buffer (pH 7.4) before incubating into different temperatures (40–70 °C) in 15 min. The remaining P450 content was recorded by the CO‐difference spectrum after centrifuging at   13 680 ***g*** for 15 min to remove aggregates.

Protein (5 µm) was dissolved in different pH value buffers, namely, 20 mm citrate buffer (pH 4–5), 20 mm potassium phosphate buffer (pH 6–8) and 20 mm Tris–HCl buffer (pH 8.5–9). Samples were incubated at the optimal temperature (50 °C) for 15 min, and we calculated the relative content.

### Half‐life of enzyme

The purified protein was dissolved in 20 mm potassium phosphate buffer (pH 7.4) and incubated at 50 °C. The sample was taken every 15 min for a total of 120 min and measured by CO‐difference spectra. The half‐life (*t*_1/2_) index was calculated at a required time when CYP quantity reduced 50% of integrity based on the equation *t*_1__/2_ = ln 2/*k*_d_, where *k*_d_ is the first‐order rate constants determined by linear regression of ln (residual absorption at 450 nm) versus the incubation time (*t*) [19, 20].

### Melting temperature

A melting curve was determined using far‐UV CD by JASCO J‐715 spectropolarimeter (Jasco GmbH, Gross‐Umstadt, Germany). The ellipticity was measured at 211 nm as a function of temperature in the range between 25 and 95 °C using a temperature slope of 1 °C·min^−1^, data pitch 0.1 °C. For the measurement, the concentration of the P450‐T2 was 2 µm resuspended in 10 mm potassium phosphate buffer (pH 7.4). The CD spectra were recorded between 190 and 260 nm every 10 °C.

### Redox partner screening

The capacity of the electron transfer partners was determined by comparing the peak at 450 nm of the CO‐complex T2 reduced with the different redox systems and the peak at 450 nm of the CO‐complex T2 reduced with sodium dithionite. P450‐T2 was mixed with ferredoxins from *Bacillus megaterium* (Fdx2, Fdx3) [21, 22], mammalian truncated adrenodoxin (Adx_4–108_) [23, 24] or yeast Etp1 [25] and corresponding ferredoxin reductases (BmCPR, AdR or Arh1) at a ratio of 1 : 40 : 5 (0.25 µm P450‐T2: 10 µm Fdx: 1.25 µm FdR) in 50 mm HEPES buffer (pH 7.4). NADPH was added to achieve a final concentration of 1 mm.

### Substrate binding

A list of aromatic substrates, including citrinin, l‐mimosine, piceatannol, l‐resveratrol, butein, emodin, luteolin, morin, isoscopoletin, scopoletin and nalidixic acid, were tested. The substrates were dissolved in dimethyl sulfoxide solution (10 mm DMSO) to reach final substrate concentrations of 50 or 100 mm. The reaction was quantitated at room temperature under aerobic conditions using a UV–vis scanning photometer (UV‐2101PC; Shimadzu) equipped with two tandem cuvettes based on the spin‐state shifts [26].

## Results and Discussion

### Identification and bioinformatics analysis of P450 T2

By comparison with the assigned CYP P450 sequences in the P450 homepage, P450‐T2 showed 73.15% identity with CYP203A1 from *Rhodopseudomonas palustris* and 62.92% identity with CYP203A2 from *Novosphingobium aromaticivorans*. In the phylogenetic tree, P450‐T2 formed a clade with CYP203A1 and CYP203A2, supporting the closest relation between them (Fig. S1). Therefore, we suppose that P450‐T2 is a member of the CYP203A subfamily. Multiple protein sequences alignment also showed high conservation between P450‐T2 and CYP203A1 and CYP203A2 (Fig. S2). The predicted *T*_m_ indices of P450‐T2, CYP203A1 and CYP203A2 were 1.25, 1.58 and 1.02, respectively, supporting their predicted *T*_m_s were greater than 65 °C. P450‐T2 was also predicted as an alkali‐resistant protein with a probability of 0.82 using the AcalPred tool. Therefore, P450‐T2 was selected for expression.

CYP203A1 from *R. palustris* was found to bind a broad range of substrates, in particular, substituted aromatic compounds, such as 4‐hydroxybenzoic acid, 3,4‐dichlorophenol, 1,2,4‐trichlorobenzene, 2,3,5‐trichlorobenzoic acid and pentachlorobenzene [27]. Its crystal structure has been resolved [28]. The CYP203A2 from *N. aromaticivorans* also showed binding of aromatic compounds [29]. Therefore, we hypothesized that P450‐T2 might be able to bind to aromatic compounds.

### Expression and purification of recombinant P450‐T2

**Fig. 1:**
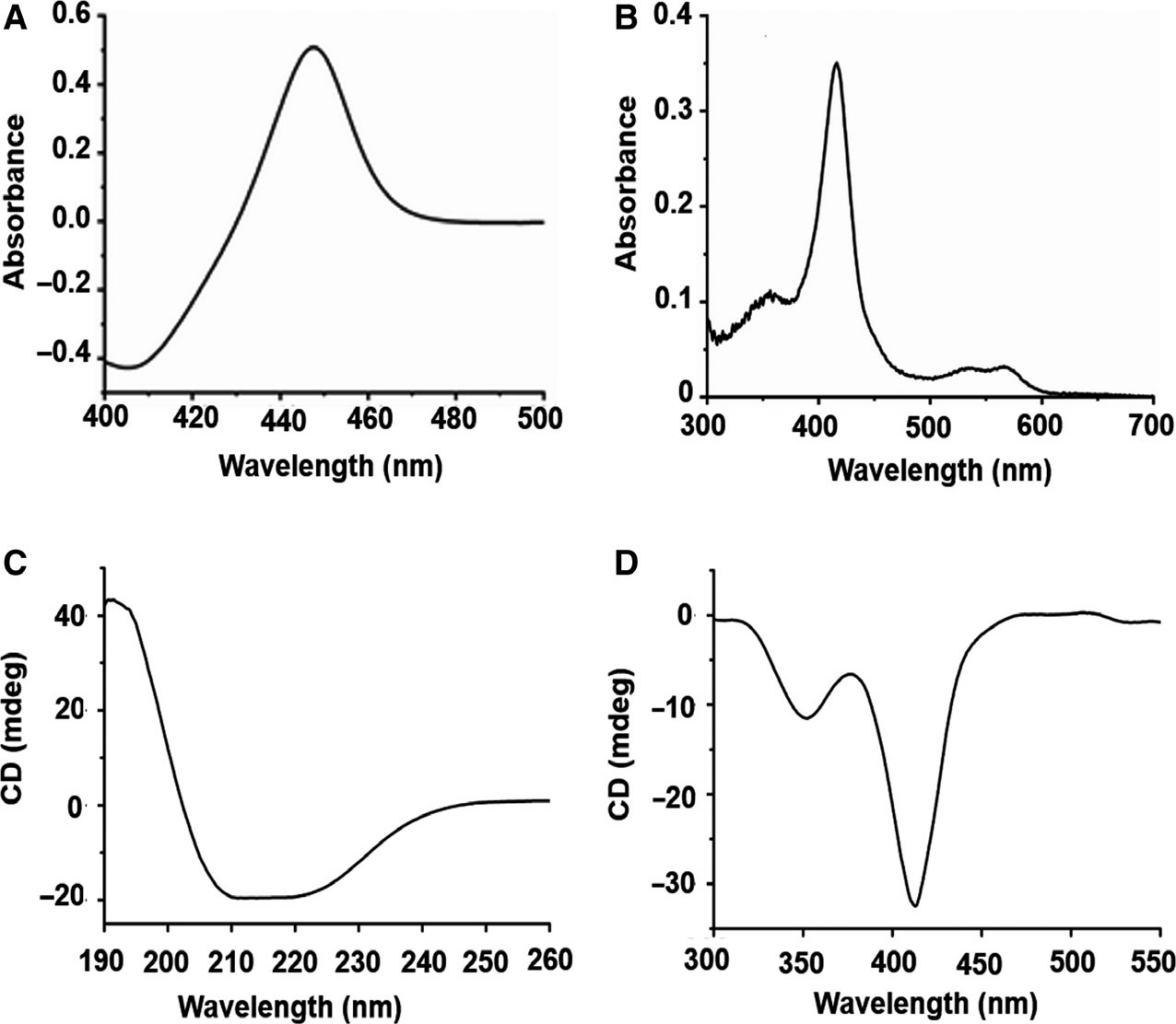
Spectral characteristics of P450‐T2. (A) The Fe^II^‐CO complex of P450‐T2 showed a maximal absorption at 450 nm. (B) UV‐Vis spectroscopy of the purified P450‐T2. CD spectra of P450‐T2 in the far‐UV (C) and near‐UV‐Vis (D), which were recorded with 4 and 20 µm enzyme, respectively, in 10 mm potassium phosphate buffer (pH 7.4) at 25 °C.

The recombinant P450‐T2 was expressed in different *E. coli* strains, including *E. coli* BL21(DE3), *E. coli* JM109(DE3) and *E. coli* C43(DE3) with a yield of 241.2, 124.7 and 514.0 nmol P450‐T2 per liter of bacterial cell culture, respectively (Fig. S3). The *E. coli* C43(DE3) carrying the pET17‐T2 vector expressed the highest yield (514.0 nm). CO‐difference spectral analysis of P450‐T2 showed the maximal absorption of the Soret peak at 450 nm without any peak at 420 nm. It implied that P450‐T2 was expressed in an active form (Fig. 1A). Purification of P450‐T2 was performed with an IMAC‐Ni^2+^ column in a one‐step procedure. The molecular weight of the purified P450‐T2 was estimated to be 44.3 kDa by SDS/PAGE as predicted (Fig. S3).

### UV‐Vis absorption spectroscopy

As a heme‐binding monooxygenase, the purified P450‐T2 displayed the major Soret (γ) band at 417 nm (Fig. 1B) and the two minor α and β bands at 568 and 537 nm, respectively, in the low‐spin state, indicating the ferric aqua‐ligand bound P450 resting state [30]. Moreover, the far‐UV CD spectrum of P450‐T2 revealed a negative dichroic double band with minima at 208 and 220 nm, presenting the conformation of both α‐helices and β‐sheets in terms of secondary structure (Fig. 1C). In contrast, two negative bands, both at the delta (peak at 350 nm) and the Soret (peak at 408 nm) region, were observed in the near‐UV and visible region (Fig. 1D), suggesting the negative cotton effect. These characteristics were consistent with features of other CYP P450s [30, 31].

### Thermal stability

**Fig. 2:**
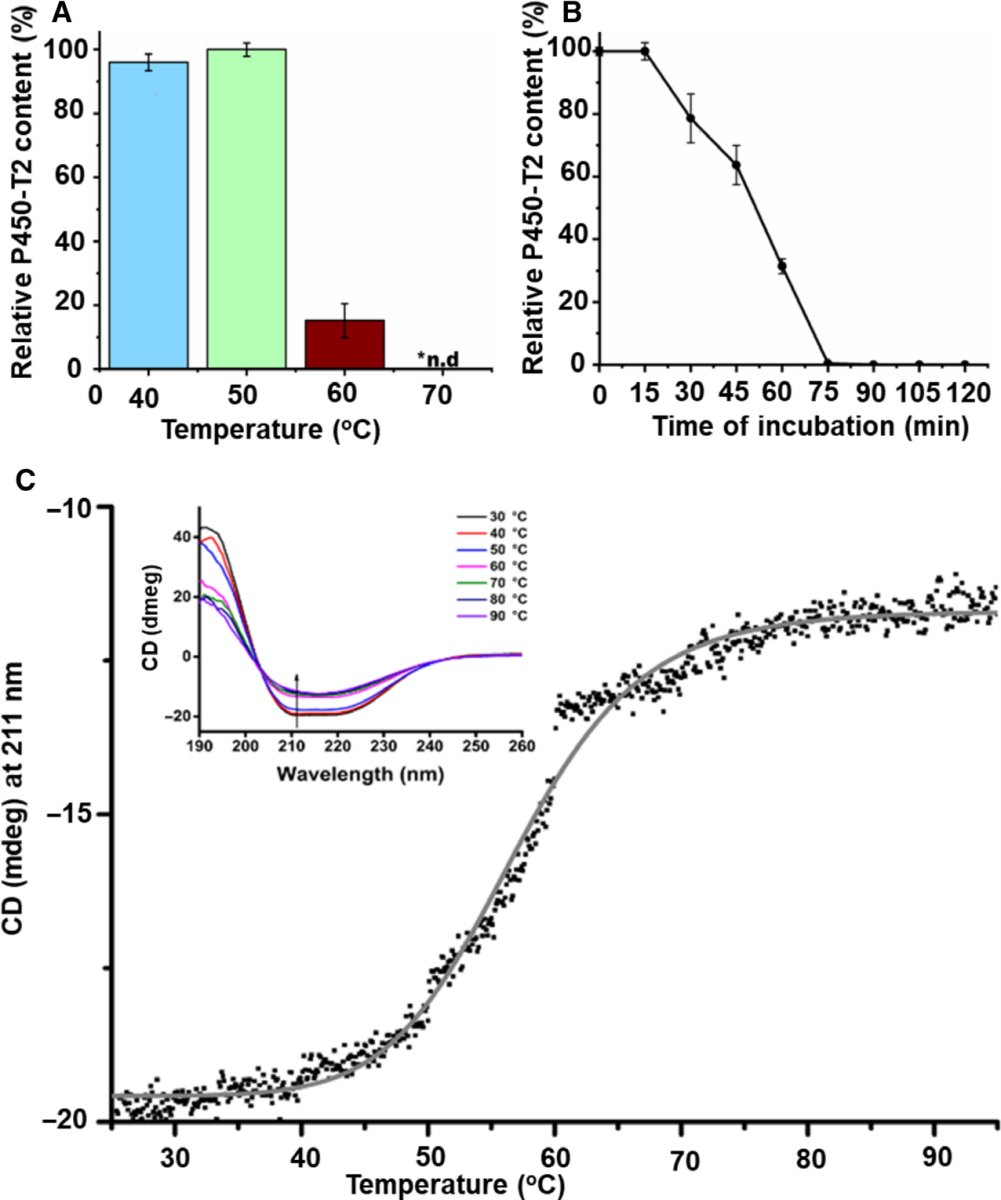
Thermal stability of P450‐T2. (A) Measurement of the optimal temperature of P450‐T2. The experiments were conducted at 40, 50, 60 and 70 °C. (B) Alteration of P450‐T2 integrity when incubating at 50 °C from 0–120 min. Samples were collected every 15 min, and the CO‐difference spectra were measured. The error bars in (A) and (B) represent the standard deviation across three independent replicates (*n* = 3). (C) Melting point of P450‐T2. The ellipticity was measured at 211 nm as a function of the temperature in the range between 25 and 95 °C using a temperature slope of 1 °C·min^−1^, data pitch 0.1 °C. The data points were plotted and fitted (gray solid line) with originpro 9.0G program (OriginLab, Northampton, MA, USA). For the measurement, the concentration of the P450‐T2 was 2 µm resuspended in 10 mm potassium phosphate buffer (pH 7.4). The inset shows the CD spectra of the P450‐T2 in the far‐UV region at the indicated temperatures (arrow shows the direction of the peak by increasing the temperature at 211 nm). The CD spectra were recorded between 190 and 260 nm every 10 °C.

Thermal stability is attracting much attention for enzyme application in the biotechnological and pharmaceutical industry. After incubation of P450‐T2 at four different temperatures (40, 50, 60 and 70 °C), the highest integrity was observed at 50 °C (Figs 2A and S4), which was similar to the optimal temperature of CYP154H1 obtained from the thermophile soil bacterium *Thermobifida fusca* [32]. At 40 °C, the P450‐T2 content was nearly similar to that at 50 °C; however, a small peak at 420 nm indicated an inactive form of the protein (Fig. S4). P450‐T2 was completely degraded at the temperatures higher than 60 °C (Fig. 2A). Most P450s that are obtained naturally show a moderate temperature optimum under 40 °C [8], except for a limited number of extreme thermophilic P450s, such as CYP119A1 and CYP175A1, which derived from archaea and showed an optimal temperature at 70 and 87 °C, respectively [33, 34].

P450‐T2 was incubated for 15–120 min at its optimal temperature (50 °C), and its half‐life index was found to be 50.2 min (Fig. 2B). In Fig. 2C (inset), the appearance of an intense negative minimum around 208 and 222 nm, as well as a positive maximum around 197 nm, confirmed the existence of a dominant α‐helical secondary structure [35]. At 50 °C, a little loss of magnitude of the negative of the CD signal was shown, compared with those at a temperature range of 25–45 °C (Fig. 2C). The *T*_m_ was calculated as 56.8 ± 0.08 °C (*R* ^2^ = 0.99).

Compared with other recently discovered thermostable CYP P450s, such as CYP119, CYP175A1, CYP154H1 and CYP231A2, which showed *T*_m_ values at 90 °C [36], 80 °C [37], 67 °C [32] and 65 °C [38], respectively, P450‐T2 displayed a lower thermal stability. Therefore, P450‐T2 is considered a moderate thermostable enzyme. However, the thermal stability of other members of the CYP203A subfamily, e.g. CYP203A1 and CYP203A2 from *R. palustris* and *N. aromaticivorans,* respectively, has not been reported so far but would be interesting to compare with data for P450‐T2.

### Optimal pH

The pH optimum value may differ from one CYP P450 to another. In this study, P450‐T2 showed the highest amount of CO‐reduced form at pH 7.5–8.5 with a peak at pH 8.0, and the P450 content decreased dramatically at pH 9 (Fig. S5). The isoelectric point was predicted to be 6.03 using the corresponding tool of Expasy (https://web.expasy.org/compute_pi/), whereas those of CYP203A1 (*R. palustris*) and CYP203A2 (*N. aromaticivorans*) were 5.82 and 5.64, respectively. Along with other physical conditions, the environmental pH may affect enzyme activity and bacterial growth [39]. The Binh Chau hot spring (10°36′05.0″N and 107°33′33.5″E) is a slightly alkaline hot spring with an environmental pH of 7.4. Thus, the microbial community there may produce enzymes that increased stability under slightly alkaline conditions and help the cells on adapting well to its local environment.

### Investigation of electron transfer partners

**Fig. 3:**
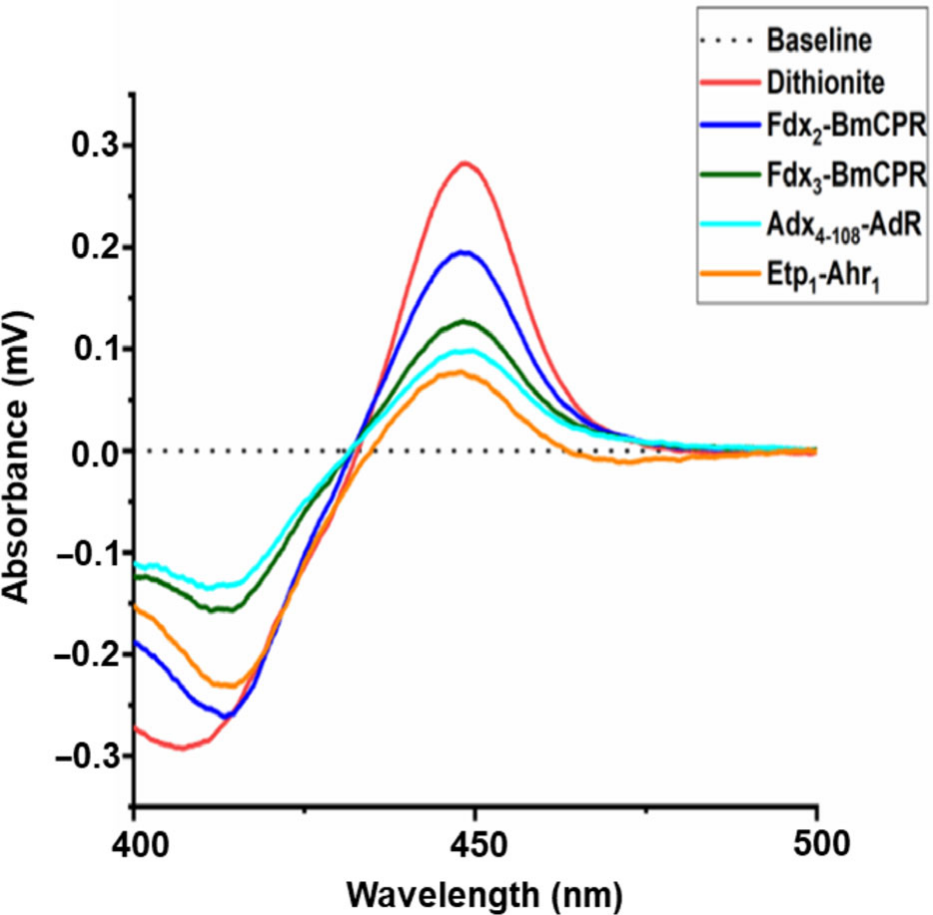
Screening of electron transfer partners for P450‐T2. The dithionite reduced CO‐difference spectrum (red line) was compared with the CO‐complex spectrum after reduction by truncated Adx_4–108_–AdR (cyan line), Etp_1_–Arh_1_ (orange line), Fdx2–BmCPR (dark green line) and Fdx3–BmCPR (blue line). The baseline is performed in a black dotted line. A 1 mL mixture of CYP : ferredoxin : ferredoxin reductase of 1 : 40 : 5 (0.25 µm P450‐T2: 10 µm Fdx : 1.25 µm FdR) in 50 mm HEPES buffer (pH 7.4). NADPH solution (1 mm) was supplemented for starting the reduction.

As an external monooxygenase, P450‐T2 requires electron transfer partners to activate molecular oxygen and perform the conversion of substrates. Because we were not able to find the natural redox partner sequences for P450‐T2 in the DNA metagenomic database, we reconstituted P450‐T2 with different ferredoxin/ferredoxin reductases from mammalian (Adx_4–108_–AdR), yeast (Etp_1_–Arh_1_) and *B. megaterium* (Fdx_2_–BmCPR, Fdx_3_–BmCPR) systems. Based on the spectrum of the reduced CO‐complexed enzyme of P450‐T2, the effects of a range of redox partners in the first electron transfer were compared (Fig. 3). P450‐T2 was reduced rapidly by sodium dithionite, whereas in the other samples, the enzyme spent around 4–5 min to reach the maximal peak at 450 nm in an aerobic environment. All redox partners in this study showed a reduction of P450‐T2. However, the redox system BmCPR‐Fdx2 exhibited the highest efficiency, with 60% of peak recovery as compared with dithionite reduced CO‐difference peak. The redox partners BmCPR and Fdx2 originated from *B. megaterium* DAM319 [40] and have been shown to support efficiently the activity of CYP106A1 [21] and CYP107DY1 [22].

### Substrate spectrum screening

**Fig. 4:**
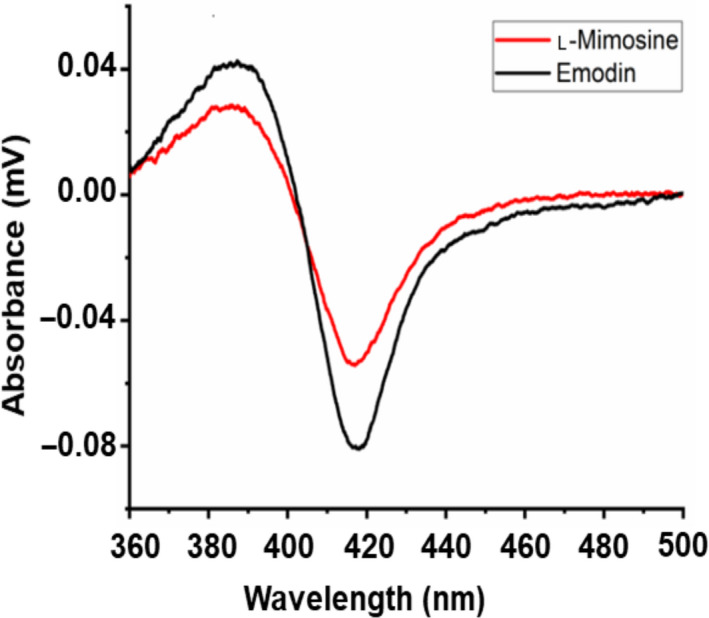
Type I shift induced by binding of l‐mimosine (red line) and emodin (black line) to P450‐T2. Substrates (50 mm) were dissolved in dimethyl sulfoxide before mixing with solution of P450‐T2 (3 μM) in 10 mm potassium phosphate buffer (pH 7.4) supplemented with 20% glycerol.

After identifying effective redox partners of the thermophilic P450‐T2, it was of interest to obtain information about potential substrates of this enzyme. To date, limited information is available concerning the CYP203A subfamily. However, CYP203A1 from *R. palustris* may play a pivotal role in metabolic pathways of aromatic ring degradation [27, 28]. Therefore, P450‐T2 was expected to be able to bind to aromatic compounds. Eleven phenolic compounds listed in Fig. S6 were screened concerning their binding capacities to the active site of P450‐T2 spectrophotometrically, where the displacement of the sixth ligand to the heme iron by the substrate can be followed. Substrate binding results in a shift from low spin toward the high spin of the ferric heme iron (type I shift) showing a minimum Soret absorption around 420 nm and a maximum at about 390 nm [30]. Interestingly, P450‐T2 displayed a clear type I shift with l‐mimosine and emodin (Fig. 4). l‐mimosine may also have anti‐inflammatory activity and an inhibitory effect on tumor necrosis factor‐alpha and interleukin‐6 generation [41]. In contrast, emodin is an anthraquinone derivative, which is produced by some fungal species belonging to the *Aspergillus*, *Penicillium* and *Talaromyces* genera [42, 43]. This implies that P450‐T2 may become a good candidate for pharmaceutical application. Further studies analyzing the conversion of the putative substrates and the identification of the formed products are needed to better define the function and possibilities of application of this CYP P450. Nevertheless, our findings have expanded the substrate binding range of thermophilic CYP P450s and their potential for the conversion of aromatic compounds.

## Conclusions

In this paper, we identified, expressed, purified and characterized a thermophilic CYP P450, named P450‐T2, which shared 73.15% amino acid sequence identity with the CYP203A1 from *R. palustris*. The recombinant protein gained the highest yield in *E. coli* C43(DE3) strain with 541 nm. The molecular weight of the purified enzyme was estimated at 44.3 kDa. Biophysical and biochemical properties of P450‐T2 revealed that P450‐T2 is a moderate thermophilic enzyme. Ferredoxin and ferredoxin reductase obtained from *B. megaterium* were the most suitable heterologous redox partners for P450‐T2. Several aromatic compounds, such as l‐mimosine and emodin, were demonstrated to be promising substrates of P450‐T2, suggesting P450‐T2 may be used in pharmaceutical approaches.

## Conflict of interest

The authors declare no conflict of interest.

## Author contributions

K‐TN, RB, MM and H‐HN conceived and designed the experiments. K‐TN, T‐T‐X‐L, T‐H‐N‐L and T‐T‐M‐P performed the experiments. V‐TN analyzed software. K‐TN and MM analyzed data. K‐TN, N‐LN, H‐HN and RB curated data. K‐TN and N‐LN wrote the manuscript. K‐TN and RB reviewed and edited the manuscript.

## Supporting information


**Fig. S1.** Phylogenetic tree showing the position of P450‐T2 in the CYP203 family and the closest CYP P450s. The evolutionary history was inferred using the maximum likelihood method and LG model.
**Fig. S2.** Multiple alignment of P450‐T2 and CYP203A1 and CYP203A2.
**Fig. S3.** Expression and purification of P450‐T2. In the left, the heterologous expression of P450‐T2 in *E. coli* BL21(DE3) (soft beige color), *E. coli* JM109(DE3) (fawn color) and *E. coli* C43(DE3) strains (orange color). The error bars represent the standard deviation across three independent replicates (*n* = 3). The expression was performed in 2‐L baffled flasks containing 250 mL TB medium, which was induced by 1 mm IPTG and 0.5 mm δ‐aminolevulinic acid at 30 °C, 150 r.p.m. for 48 h. SDS/PAGE of total lysate from *E. coli* C43(DE3) carrying pET17b‐T2 vector (lane 1), purified P450‐T2 (lane 2) and precision marker (Bio‐Rad) (lane M) is presented in the right.
**Fig. S4.** Effect of temperature on P450‐T2 content. Purified enzyme (5 µm) was dissolved in 20 mm potassium phosphate buffer (pH 7.4), then incubated at different temperatures (40–70 °C) for 15 min. P450‐T2 displayed the best integrity at 50 °C, whereas its content lost at the temperatures higher than 60 °C. At 40 °C, a small peak at 420 nm indicated an inactive form of protein along with a maximum peak at 450 nm.
**Fig. S5.** Effect of pH on P450‐T2 content. Purified enzyme (5 µm) was dissolved in different buffers, including 20 mm citrate buffer (pH 4–5), 20 mm potassium phosphate buffer (pH 6–8) and 20 mm Tris–HCl buffer (pH 8.5–9). The error bars represent the standard deviation across three independent replicates (*n* = 3).
**Fig. S6.** List of the selected substances for screening the putative substrates of P450‐T2.

## Data Availability

The raw data are available from the corresponding author upon reasonable request.
