# Supplementary material for: Characterization of a thermophilic cytochrome P450 of the CYP203A subfamily from Binh Chau hot spring in Vietnam
Source: FEBS Open Bio. 2020 Nov 30;11(1):124–32. doi: 10.1002/2211-5463.13033 (PMC7780096; doi:10.1002/2211-5463.13033)
Supplement: Supplementary file 1 — Fig. S1. Phylogenetic tree showing the position of P450‐T2 in the CYP203 family and the closest CYP P450s. The evolutionary history was inferred using the maximum likelihood method and LG model. Fig. S2. Multiple alignment of P450‐T2 and CYP203A1 and CYP203A2. Fig. S3. Expression and purification of P450‐T2. In the left, the heterologous expression of P450‐T2 in E. coli BL21(DE3) (soft beige color), E. coli JM109(DE3) (fawn color) and E. coli C43(DE3) strains (orange color). The error bars represent the standard deviation across three independent replicates (n = 3). The expression was performed in 2‐L baffled flasks containing 250 mL TB medium, which was induced by 1 mm IPTG and 0.5 mm δ‐aminolevulinic acid at 30 °C, 150 r.p.m. for 48 h. SDS/PAGE of total lysate from E. coli C43(DE3) carrying pET17b‐T2 vector (lane 1), purified P450‐T2 (lane 2) and precision marker (Bio‐Rad) (lane M) is presented in the right. Fig. S4. Effect of temperature on P450‐T2 content. Purified enzyme (5 µm) was dissolved in 20 mm potassium phosphate buffer (pH 7.4), then incubated at different temperatures (40–70 °C) for 15 min. P450‐T2 displayed the best integrity at 50 °C, whereas its content lost at the temperatures higher than 60 °C. At 40 °C, a small peak at 420 nm indicated an inactive form of protein along with a maximum peak at 450 nm. Fig. S5. Effect of pH on P450‐T2 content. Purified enzyme (5 µm) was dissolved in different buffers, including 20 mm citrate buffer (pH 4–5), 20 mm potassium phosphate buffer (pH 6–8) and 20 mm Tris–HCl buffer (pH 8.5–9). The error bars represent the standard deviation across three independent replicates (n = 3). Fig. S6. List of the selected substances for screening the putative substrates of P450‐T2. [file FEB4-11-124-s001.pdf]

SUPPLEMENTARY MATERIALS

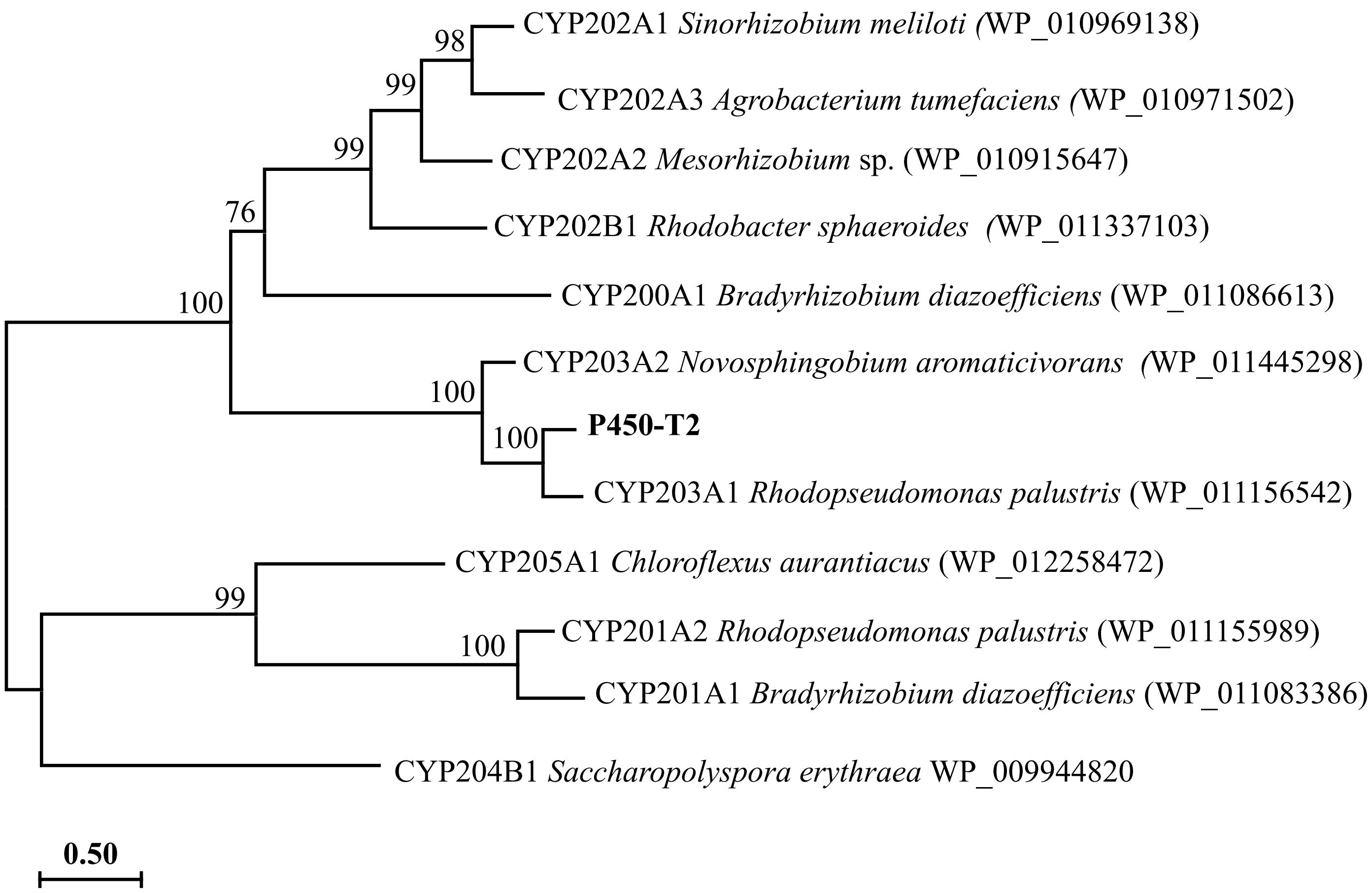

Fig. S1. Phylogenetic tree showing the position of P450-T2 in the CYP203A subfamily and the closest cytochrome P450s. The evolutionary history was inferred using the maximum likelihood method and LG model.

```
CLUSTAL O(1.2.4) multiple sequence alignment


P450-T2         ----MGLGSFHFDPYSPAIDADPFPSYKRLRDEFPCFWSEEAQMWILSRYSDIVTAGQDW    56
CYP203A1        --------MFSFDPYSPIVDADPFPLYKTLRDEYPVFWSEPAQMWILSRYLDVAGAGSNW    52
CYP203A2        MATVIERPQFRFDPYSPAIDADPFPAYKVLRDEYPCFWSEEAGKWVLSRYDDVLAALQDW    60
                         * ****** :****** ** ****.* **** *  *.**** *.  * .:*

P450-T2         QTYSSASGNLMTELPGRAGATLGSSDPPKHDRLRGLIQHAFMKRNLMALEEPIRDVAKQV    116
CYP203A1        QVFSSAKGNLMTELPNRAGATLGTTDPPRHDRLRGLVQHAFMKRNLEALAEPMREIARDA    112
CYP203A2        RTYSSAKGNLVDEFPGRAGSTLGSSDPPRHDRLRALIQSAVTKRALEHIIAPARASAQAH    120
                :.:***.***: *:*.***:***::***:*****.*:* *. ** *  :  * *  *:

P450-T2         FAQVKGVKEFDFK-DVSSQFTVKVLMAALGLPMGEDALVPEHEVRENAVLMVQSDARTRA    175
CYP203A1        AEALRGRDQFDFISDFSSKFTVRVLFAALGLPMGDEQ-----TVRDKAVLMVQSDPVTRA    167
CYP203A2        LAALADKPVFDLVGDYTSKLTVDLLFYLFALPDEGAQ-----QVRENAVLMVQTDPVTRQ    175
                   : .   **:  * :*::** :*:  :.**          **::******.:*  **

P450-T2         KGPEHIAAYNWMQDYASKVIAMRRASPQNDLISNFALAEIDGDRLDDREVLLTTTTLIMA    235
CYP203A1        KGPEHLAAYAWMQDYASSVIAQRRAEPKNDLISHFSMAEIDGDRLDEREVLLTTTTLIMA    227
CYP203A2        KSPEHLAAFHWMADYAEKLVASRKANPGDDLLSSFITAEIDGEKLLDKEVQLTVTTLIMA    235
                *.***:**: ** ***..::* *:*.* :**:* *  *****::* ::** **.******

P450-T2         GVESLGGFMMMFAYNLATFDEARRAVVANPALLPDAIEESLRFNTSAQRFRRRLMKDVTL    295
CYP203A1        GIESLGGFMSMLALNLADFADARRAVVADPALLPDAVEESLRYNTSAQRFKRCLQSDLTL    287
CYP203A2        GIESLSGFMAMFGLNLADYPEARSALVADPSLIPDAIEESLRFNTSAQRFKRTLTRDVEL    295
                *:***.*** *:. *** : :** *:**:*:*:***:*****:*******:* *  *: *

P450-T2         HGQTMKEGDFVCLAYGSGNRDERQYPNPDVYDIARKPRGHLGFGGGVHACLGTAIARLAV    355
CYP203A1        HGVTMKAGDFVCLAYGSANRDERQFPNPDVYDVKRKPKGHLGFGGGVHACLGSAIARMAI    347
CYP203A2        HGQVMKAGDAVILAYGSANRDERMFENPDVYDITRKPRRHLGFGGGVHACLGSMIGRLAT    355
                **  .** ** * *****.***** : ******: ***: *************: *.*:*

P450-T2         KIAFEEFHQVVPDYRRVADQLPWMPSSTFRSPLVLQLKAQ--------  395
CYP203A1        RIAFDEFHKVVPDYTRTEQQLNWMPSSTFRSPLRLDFAVEQAASRSAA  395
CYP203A2        QIAYEELLKAVPDFRRADAPLDWVPSSNFRSPKSLMLEKKA-------  396
                :**::*: :.***: *.   * *:***.****  * :  :
```

Fig. S2. Multiple alignment of P450-T2 and CYP203A1 and CYP203A2.

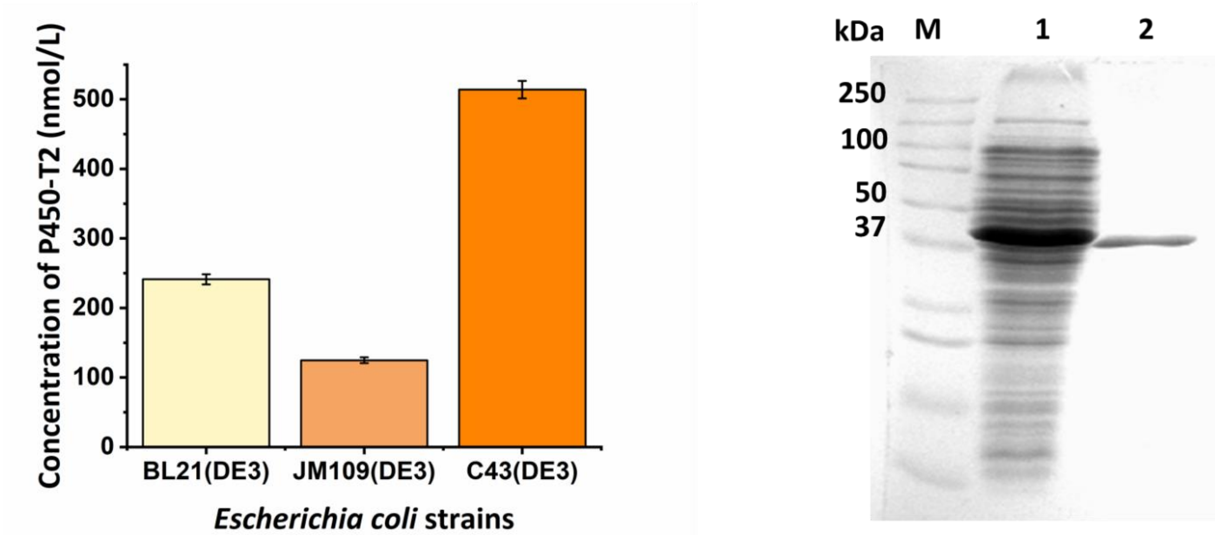

Fig. S3. Expression and purification of P450-T2. In the left, the heterogous expression of P450-T2 in *E. coli* BL21(DE3) (soft beige colour), *E. coli* JM109(DE3) (fawn colour) and *E. coli* C43(DE3) strains (orange colour). The error bars represent the standard deviation across three independent replicates (*n*=3). The expression was performed in 2-L baffled flasks containing 250 mL TB medium which was induced by 1 mM IPTG and 0.5 mM δ-Ala at 30 ^{o}C, 150 rpm for 48 h. SDS-PAGE of total lysate from *E. coli* C43(DE3) carrying pET17b-T2 vector (lane 1), purified P450-T2 (lane 2) and precision marker (Biorad) (lane M) is presented in the right.

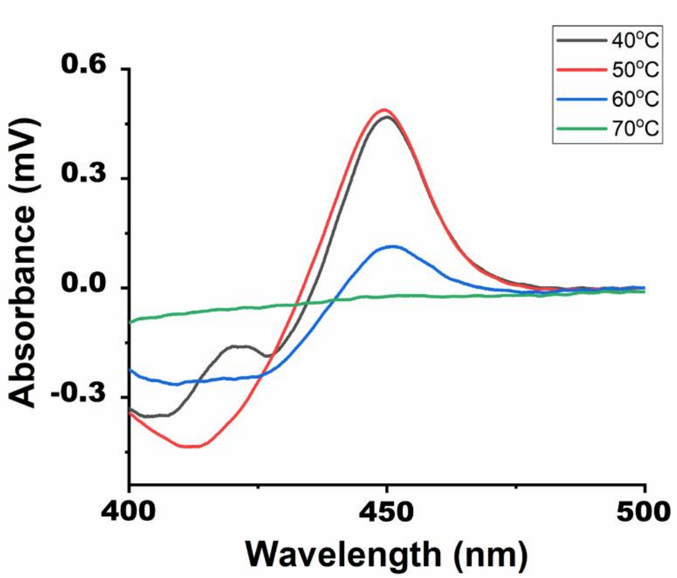

Fig. S4. Effect of temperature on P450-T2 content. Purified enzyme (5 µM) was dissolved in 20 mM potassium phosphate buffer pH 7.4, then incubated at different temperatures (40-70 °C) for 15 min. P450-T2 displayed the best integrity at 50 ^{o}C, whereas its content lost at the temperatures higher than 60 ^{o}C. At 40 ^{o}C, a small peak at 420 nm indicated an inactive form of protein along with a maximum peak at 450 nm.

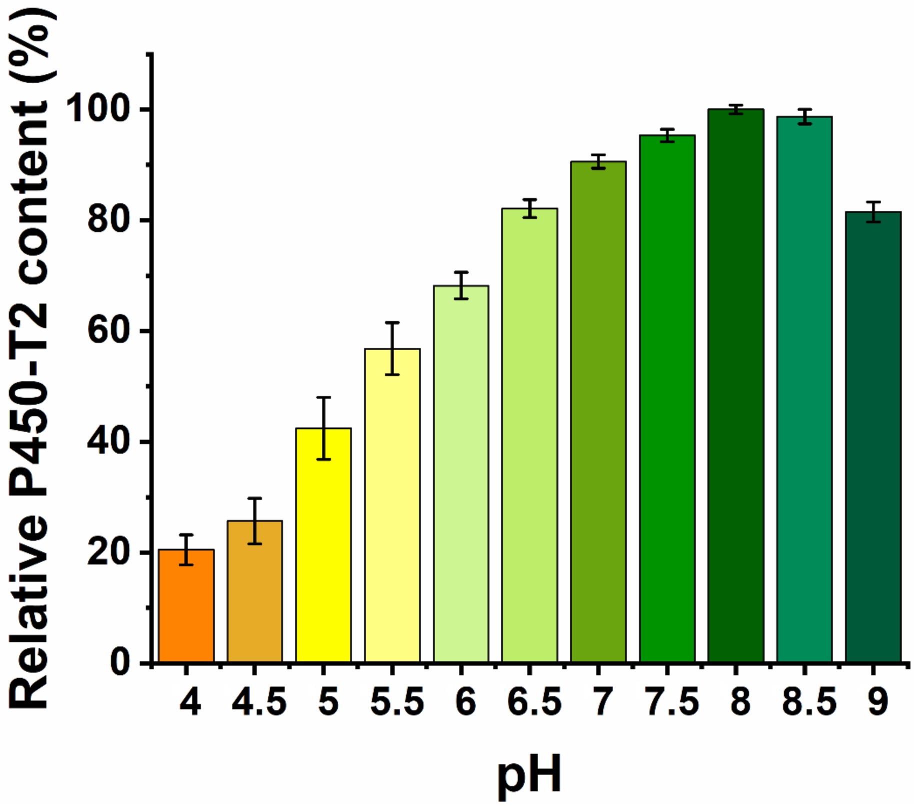

Fig. S5. Effect of pH on P450-T2 content. Purified enzyme (5 μM) was dissolved in different buffers, including 20 mM citrate buffer (pH 4-5), 20 mM potassium phosphate buffer (pH 6-8), and 20 mM Tris/HCl buffer (pH 8.5-9). The error bars represent the standard deviation across three independent replicates (*n*=3).

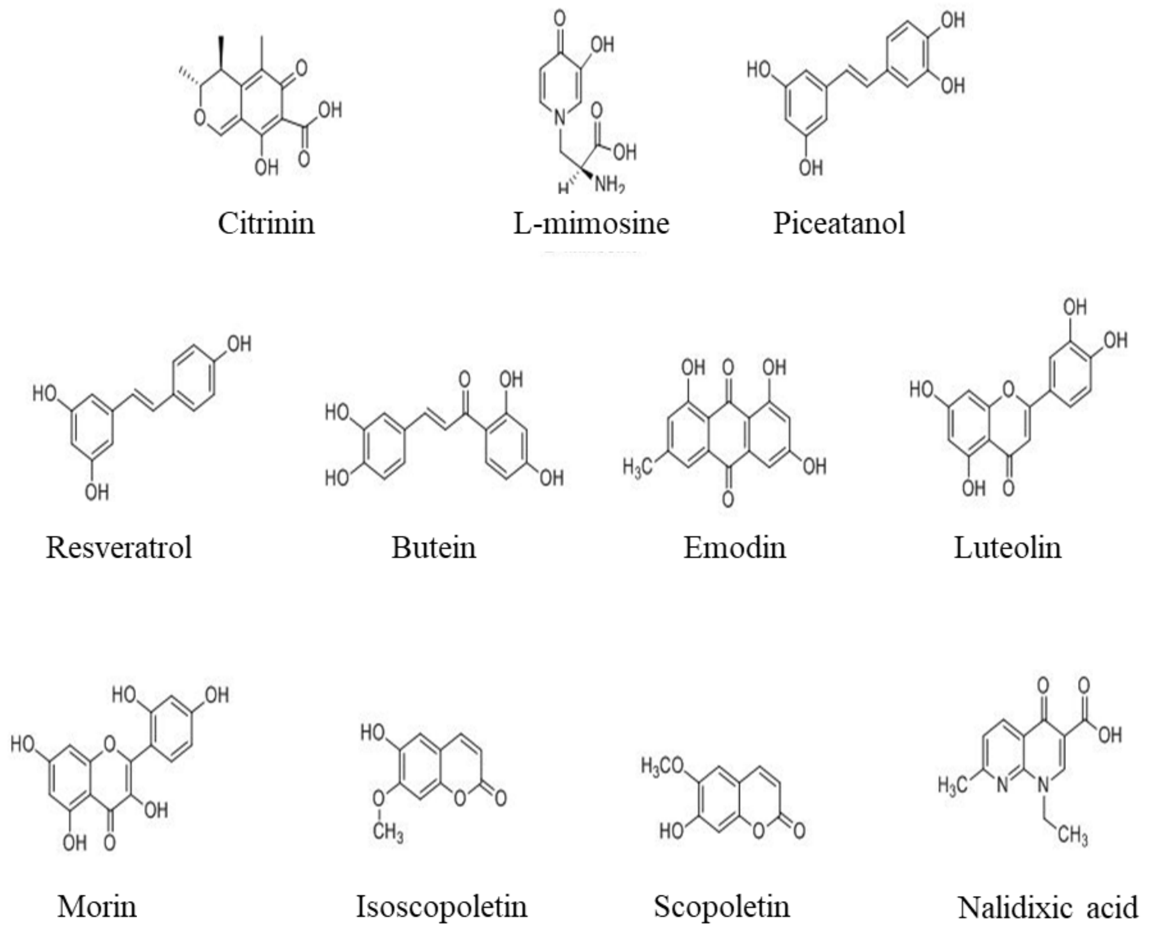

Fig. S6. List of the selected substances for screening the putative substrates of P450-T2.
